# Challenges and opportunities in managing biodegradable plastic waste: A review

**DOI:** 10.1177/0734242X241279902

**Published:** 2024-09-30

**Authors:** Namrata Mhaddolkar, Thomas Fruergaard Astrup, Alexia Tischberger-Aldrian, Roland Pomberger, Daniel Vollprecht

**Affiliations:** 1 Chair of Waste Processing Technology and Waste Management (AVAW), Montanuniversität Leoben (MUL), Leoben, Austria; 2 DTU SUSTAIN, Department of Environmental Engineering, Danish Technical University (DTU), Lyngby, Denmark; 3 Ramboll, Copenhagen S, Denmark; 4 Chair of Resource and Chemical Engineering, University of Augsburg, Augsburg, Germany

**Keywords:** Biodegradable plastic waste management, waste collection and sorting, consumer confusion, recycling, EU legislation, source separation guidelines

## Abstract

Biodegradable plastics have certain challenges in a waste management perspective. The existing literature reviews fail to provide a consolidated overview of different process steps of biodegradable plastic waste management and to discuss the support provided by the existing legislation for the same. The present review provides a holistic overview of these process steps and a comprehensive relative summary of 13 existing European Union (EU) laws related to waste management and circular economy, and national legislations plus source separation guidelines of 13 countries, to ensure the optimal use of resources in the future. Following were the major findings: (i) numerous types and low volumes of biodegradable plastics pose a challenge to developing cost-effective waste management infrastructure; (ii) biodegradable plastics are promoted as food-waste collection aids, but consumers are often confused about their proper disposal and are prone to greenwashing from manufacturers; (iii) industry-level studies demonstrating mechanical recycling on a full scale are unavailable; (iv) the existing EU legislation dealt with general topics related to biodegradable plastics; however, only the new proposal on plastic packaging waste and the EU policy framework for bioplastics clearly mentioned their disposal and (v) clear disparities were observed between disposal methods suggested by national legislation and available source separation guidelines. Thus, to appropriately manage biodegradable plastic waste, it is necessary to develop waste processing and material utilization infrastructure as well as create consumer awareness. In the end, recommendations were provided for improved biodegradable plastic waste management from the perspective of systemic challenges identified from the literature review.

## Introduction

With increasing ambitions for improved recycling and material circularity, the growing quantities and variability in waste materials are challenging. As part of the European Union Circular Economy Action Plan, 2020 (2020), recycling is now measured using the actual recycling rate rather than the collection rate (Pires et al., 2019). Hence, along with collecting waste, reprocessing it into new raw materials is equally important. This illustrates the importance of the waste management system for the circular economy in the European Union (EU) and implies that this system needs to be appropriately equipped for the efficient recycling of new material types introduced in products. Bioplastics are one of the examples for which this is currently not the case.

Bioplastics are defined as either bio-based or biodegradable or both (European Bioplastics, 2018). They make up 1% of entire plastic production (Magalhães Júnior et al., 2021) and are forecasted to grow dynamically in the future (Döhler et al., 2022; Hottle et al., 2013; Niaounakis, 2019), with a global production capacity of 6.3 million tonnes in 2027 (European Bioplastics e.V., 2022). Bioplastics are often promoted as environmentally friendly alternatives to conventional plastics (Castro-Aguirre et al., 2016; Cucina et al., 2021; Karan et al., 2019; Spierling et al., 2018; Thakur et al., 2018). The Intergovernmental Panel on Climate Change (2018) reported that using bioplastic could aid in maintaining a 1.5°C temperature rise. However, even with their presumed environmental friendliness (Rajvanshi et al., 2023; RameshKumar et al., 2020), bioplastics represent considerable challenges (Åkesson et al., 2021). It is acknowledged that bioplastic production should not compete with food production (Kabir et al., 2020); thus, research into raw materials from algae or agricultural-, bio-and other waste is growing (Ali et al., 2022; Chidambarampadmavathy et al., 2017; Choudhary et al., 2022; Dutt Tripathi et al., 2021; Sharma and Jain, 2020; Zanchetta et al., 2021). Although some bioplastics exhibit similar properties as conventional plastics and are biodegradable (Asgher et al., 2020; Aversa et al., 2021; Moshood et al., 2022b), they also involve more extensive processing at higher costs (Aversa et al., 2021; Lamberti et al., 2020; Sid et al., 2021). Thus, making it essential to derive value from their waste. It is recognized that bioplastics are not a panacea for plastic pollution and littering, and require proper waste management (Gisi et al., 2022; Hottle et al., 2013; Yadav and Hakkarainen, 2021). Although the bio-based non-biodegradable plastics can be processed in the conventional plastic recycling streams, the biodegradable plastics need a dedicated infrastructure, which could prove to be comparatively more challenging (Abraham et al., 2021; Soroudi and Jakubowicz, 2013).

A wide range of reviews in literature have addressed biodegradable plastic waste management and could be grouped into one of the following four categories: (i) *Sustainability, circular economy, and general waste management* – e.g., Moshood et al. (2022a) and Rai et al. (2021) explored the contribution and application of biodegradable plastics to sustainable development; (ii) *Life cycle assessment* – e.g., Hottle et al. (2013) and van Roijen and Miller (2022) investigated life cycle assessments (LCAs) related to biodegradable plastics; (iii) *Recycling of a particular biodegradable plastic* – e.g., Badia and Ribes-Greus (2016) on polyhydroxyalkanoates (PHAs) or Jamshidian et al. (2010) and Karamanlioglu et al. (2017) on polylactic acid (PLA); (iv) *Specific end-of-life methods of biodegradable plastics* – e.g., Feghali et al. (2020) discussed chemical recycling, Briassoulis et al. (2021) and Afshar et al. (2024) discussed organic recycling, whereas Kumar et al. (2023) conducted a review on mechanical, chemical and organic recycling. The detailed list of the grouped existing reviews is provided in Figure 2(b). However, none of these reviews offer a systemic evaluation of the different process steps involved in biodegradable plastic waste management, and the insights are fragmented with little overview provided.

Although evaluating on a systems level, the legislation also plays a crucial role. Thus, it is also important to understand whether existing EU laws and national legislation provide the necessary support for biodegradable plastic waste management. Although Briassoulis et al. (2019) briefly discussed certain EU regulatory frameworks for post-consumer plastics (including bioplastics), research is scarce on the applicability of existing legislation for biodegradable plastics waste management.

Thus, the existing literature reviews on biodegradable plastics do not provide a consolidated overview of the challenges in different process steps of biodegradable plastic waste management and the support provided by the prevailing legislation for the same. To ensure the optimal use of resources in the future and an improved basis for decision-making, a more consistent overview of the challenges and limitations associated with biodegradable plastic use and waste management is needed. Providing this missing overview is the aim of the present study, which is a systematic literature review. The main objective of this article is to provide a holistic evaluation of the challenges for biodegradable plastics in waste management, combined with a comprehensive summary of existing EU legislation and national legislation plus source separation guidelines for 13 countries. Additionally, each sub-section of the article has its aims:

- Biodegradable plastics as products – to understand which biodegradable plastic products are on the market, their applications, and the variety of different materials used.
- Collecting biodegradable plastic waste – to apprehend where biodegradable plastics end up in the collection systems, to understand how consumers perceive biodegradable plastics, to assess consumers’ awareness and to comprehend consumer behaviour.
- Waste processing and material utilization – to understand the challenges faced during processing biodegradable plastic waste when disposed of with packaging waste, biowaste and residual waste stream.
- Existing EU legislation – to discover what is mentioned about biodegradable plastics in waste management and circular economy-related EU legislation.
- Existing national legislation – to find out if the national regulations instruct about the disposal option for biodegradable plastics and compare these instructions to their respective national source separation guidelines.

**Figure 1.**
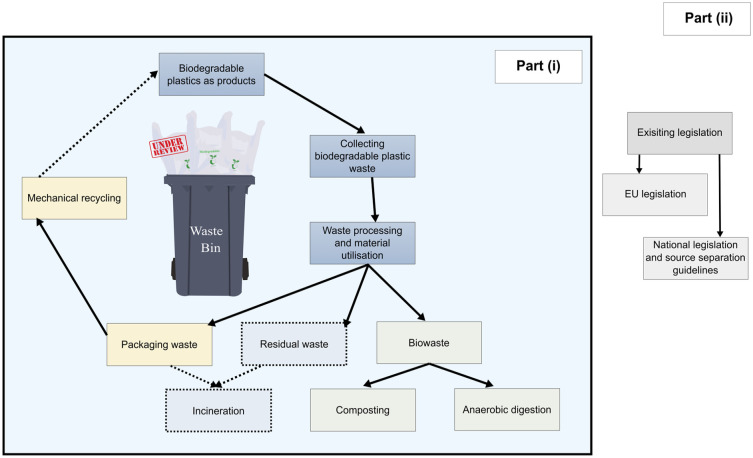
The main structure of the review article is divided into two parts: (i) managing biodegradable plastics waste and (ii) existing legislation. Part (i) has three sub-sections and Part (ii) has two sub-sections. The solid lines denote the sections included in the article, whereas the dotted lines show the exclusions.

On this basis, opportunities were discussed, and recommendations were provided for improved management of biodegradable plastics in Europe. The structural flow of the results of this review is explained in Figure 1. The methodology precedes the results section, whereas the final sections discuss the identified challenges, opportunities, recommendations and conclusion.

## Methodology

**Figure 2.**
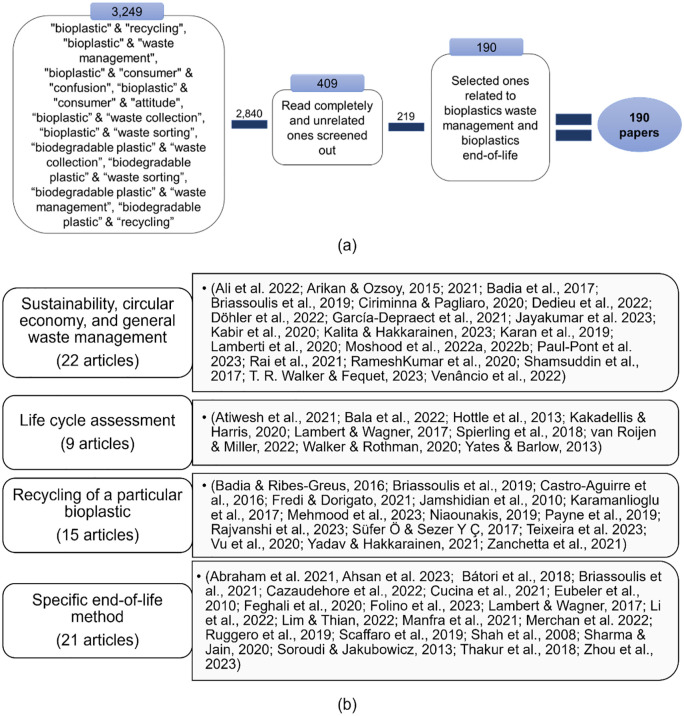
(a) Selection of research articles from literature: 190 of 3249 articles were selected. (b) Existing review articles about waste management of biodegradable plastics fall into one of the four groups.

The review has two parts: (i) managing biodegradable plastic waste and (ii) existing legislation (Figure 1). For the review of biodegradable plastics waste management (Part (i)), the keywords mentioned in Figure 2(a) were used to search literature from Google Scholar and ScienceDirect databases. This resulted in 3249 peer-reviewed journal articles, of which 2840 were rejected after partial screening for relevance to biodegradable plastics’ waste management (Figure 2(a)). The full text of 409 accepted articles was screened, leaving 190 articles related to ‘bioplastics waste management’ and/or ‘bioplastics end-of-life’. Only articles related to biodegradable plastics or bioplastics in general were considered. Of the total 190 articles, 67 articles were review articles, which were studied and grouped according to their topical focus, as illustrated in Figure 2b.

The second part of the review investigated the existing EU legislation and national regulations of 13 countries. EU legislation provides a basis and guidelines for the member states to formulate their national legislation. The 13 EU legislations related to waste management and circular economy were studied to see what they mentioned about biodegradable plastics and were categorized as ‘Waste related’, ‘Consumer related’ and ‘Others’. On the other hand, based on the study conducted by the European Parliament (2022), 13 European countries (Austria, Belgium, Denmark, Finland, France, Germany, Ireland, Italy, Lithuania, Luxembourg, Netherlands, Slovenia and Sweden) with their recycling and composting share greater than 40% were selected. For these countries, national legislation related to waste management and biowaste treatment along with source separation guidelines for consumers provided by municipalities or public responsibility organizations were studied.

## Part (i): Managing biodegradable plastic waste

### Biodegradable plastics as products

Of the total bioplastics produced, about 52% are biodegradable plastics (European Bioplastics e.V., 2022). In Table 1, the types of market-available biodegradable plastics are described. These include PLA, polybutylene adipate terephthalate (PBAT), polybutylene succinate (PBS), thermoplastic starch and their blends (European Bioplastics e.V., 2021; Scaffaro et al., 2019). Some researchers assert that starch (Laycock and Halley, 2014) and PLA (Mehmood et al., 2023) are the most suitable alternatives to conventional plastics. At the same time, numerous other kinds of biodegradable plastics are under investigation at a lab scale, which have varying structures and properties. For instance, biodegradable plastics can be produced from seaweed polysaccharides, fungal mycelium, cellulose from tree discards, chitin from crab shells and algal cellulose (Atiwesh et al., 2021; Zanchetta et al., 2021). Another example is a blend of chitosan, castor oil and yellow pumpkin starch (Hasan et al., 2018).

**Table 1.** Information about some market-available biodegradable plastics.

Type	Name	Type	Chemical structure	Calorific value (MJ/kg)	Manufacturers	Application
PLA	Polylactic acid	Bio-based biodegradable Thermoplastic	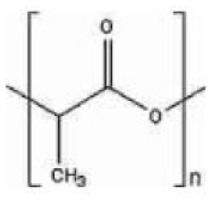	19	NatureWorks, Total Corbion, Ingeo, Futerro, Purac, Algix, BASF, Ercros, Hisun, Musashino, Synbra, Sulzer, SK Chemicals, Redici Group.	Textiles for furniture and apparel, mulch films, packaging, disposable crockery and cutlery and hygiene products.
PHB	Polyhydroxy butyrate		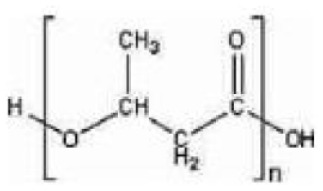	22.515	BASF, Biocycle, Bio-on, PHB Industrial, Tianan Biopolymers, Tepha.	Biomedical applications, packaging, mulch films, hygiene products, feed additive for piglets and fish.
TPS	Thermoplastic starch		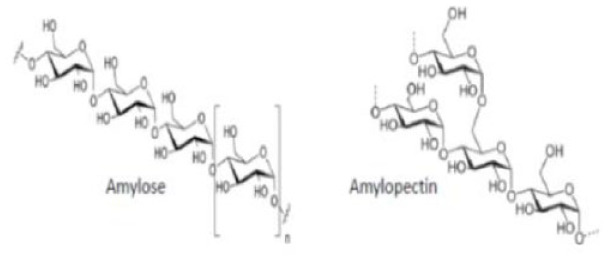	21	Agrana, BioBag, Biome, Biotec, BioLogiQ, Cardia, Futerro, Haishen, Huhtamaki, Kurarey, NatureWorks, Novamont, PSI.	Mouldable products, agricultural films, shopping bags, loose-filing foams, biowaste dustbin bags and cosmetic products.
PBS	Polybutylene succinate	Fossil-based/bio-based biodegradable Thermoplastic	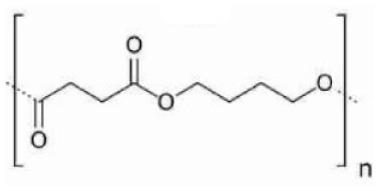	23.16	BASF, Dupont, Hexing Chemicals, IPS-CAS, IRE Chemical, Kingfa, Mitsubishi Gas Chemical, Showa, SK Chemicals, MCPP.	Food packaging, fishery, agriculture mulch films, construction, forestry, electronics, shopping bags and hygiene products.
PBAT	Polybutylene adipate terephthalate	Fossil-based biodegradable Thermoplastic	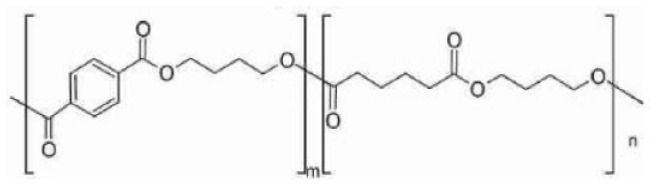	29.2	BASF, Kingfa, Tunhe, Jinfu, Xinfu, Novamont.	Packaging films, compostable dustbin bags, disposable tableware and agricultural mulch films.
PCL	Polycaprolactone		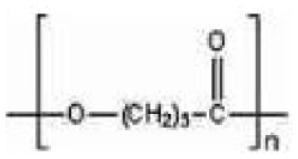	32	BASF, Perstorp.	Biomedical applications.
PVA	Polyvinyl alcohol		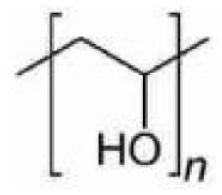	23	Nippon-Gohsei, Synthomer, Kuraray.	Biomedical applications, enhancing properties of cementitious material, agricultural products.

Source: Ali et al. (2022); Aliotta et al. (2022); Davis and Song (2006); Debuissy et al. (2017); Dedieu et al. (2022); Endres and Siebert-Raths (2011); Eubeler et al. (2010); Fredi and Dorigato (2021); Gerassimidou et al. (2021); Hermann et al. (2011b); Ioelovich (2018); Jayakumar et al. (2023); Kalita and Hakkarainen (2023); Kanwal et al. (2022); Li et al. (2022); Moshood et al. (2022a); Muniyasamy et al. (2016); Platnieks et al. (2021); Polymerdatabase (2022a, 2022b, 2022c); Rafiqah et al. (2021); Saalah et al. (2020); Schryver et al. (2010); Süfer and Sezer (2017); Wang et al. (2019); Xu et al.(2022); Zhang et al. (2022).

Packaging is one of the major applications for biodegradable plastics (Ciriminna and Pagliaro, 2020); however, there are certain limitations owing to their properties. For instance, their biodegradability sometimes hinders the optimum mechanical performance crucial for food preservation (Kakadellis and Harris, 2020; Lorite et al., 2017). They may also incur higher costs for extensive processing than conventional plastics (Aversa et al., 2021; Lamberti et al., 2020; Sid et al., 2021). Yet, it may be viable to use biodegradable plastics for short-term applications (e.g., packaging) and employ conventional plastics for long-term applications (Bala et al., 2022; Gisi et al., 2022). Moreover, their packaging applications have increased steadily since 2016, especially in single-use applications like agriculture and food-related services (Fredi and Dorigato, 2021). Thus, balancing end-of-life and long-term properties is crucial for ‘design for sustainability’ (Badia et al., 2017) and improving the marketability of these plastics requires reduced costs, increased yields, better and sustainable feedstock supplies and surety to customers about their sustainability (Iles and Martin, 2013).

### Collecting biodegradable plastic waste

Waste collection is crucial for achieving an effective biodegradable waste-management system (Abraham et al., 2021; Fredi and Dorigato, 2021; García-Depraect et al., 2021). It affects recyclate quality (Gisi et al., 2022) and the fate of biodegradable plastics (Manfra et al., 2021), ensuring the long-term recovery of recyclable products (Moshood et al., 2022a).

Separate collection aids in better treatment of the collected waste. For example, a dedicated collection system for biodegradable plastics would ease their inclusion with food waste in compost plants (Zhu and Wang, 2020). Yet, it is challenging to have a separate collection stream for them (Rahman and Bhoi, 2021) due to their variable material qualities, higher cost and lower volumes than conventional plastics, which makes them less attractive to the industry (Siltaloppi and Jähi, 2021; Wydra et al., 2021). Existing waste infrastructure may be used to collect biodegradable plastic packaging with plastic packaging waste and other biodegradable plastic products with non-packaging plastics (Gere and Czigany, 2020; Siltaloppi and Jähi, 2021). Thus, biodegradable plastics should either be collected and treated in biowaste treatment plants or sorted out of mixed plastics using costly near-infrared (NIR) sorting technology (Rujnić-Sokele and Pilipović, 2017). However, the actual source separation of these plastics is influenced by the perception of consumers about them.

#### Consumer perception about biodegradable plastics

Predominantly, it is important to understand how consumers perceive bioplastics (and biodegradable plastics) in general. Numerous qualitative research studies were focused on this area and as per their observations consumers have generally a positive inclination towards bioplastics. For instance, consumers preferred bioplastics owing to their perceived environmental benefits (Granato et al., 2022). Although some of them perceived products as sustainable based on their material type and disposal method (Boesen et al., 2019; Herbes et al., 2018; Moshood et al., 2022c), others placed more importance on raw-material origin than the end-of-life (Grebitus et al., 2020). Furthermore, biodegradable plastics were often preferred over recycled plastics (Marchi et al., 2020; Taufik et al., 2020). Table 2 summarizes key points of consumer perceptions of overall bioplastics; the articles are not specifically focused on biodegradable plastics, but they convey important points about their general outlook towards these plastics.

**Table 2.** Overview of the reviewed literature about consumer perceptions of bioplastics in general.

Authors	Country	Number of respondents	Key summary points
Lynch et al. (2017)	The Netherlands	57	Respondents, in general, were positive about these plastics. However, they expressed that their eco-friendliness should be supported by evidence and transparent information, preferably from a neutral source, such as a research institution.
Herbes et al. (2018)	France, Germany and the USA	2001	Respondents perceived a product as environment-friendly from their presumed end-of-life option.
Boesen et al. (2019)	Denmark	197	Consumers perceived the sustainability of packaging materials based on material type and disposal method rather than on production and transport impacts, contradicting the LCA results; thus, necessitating improved consumer communication.
Dilkes-Hoffman et al. (2019)	Australia	2518	Respondents perceived biodegradable plastics to have better end-of-life characteristics than recyclable plastics.
Hao et al. (2019)	China	781	Consumers attached more importance to the barrier properties, ease of use and reusability of the green packaging than their appearance and cost.
Confente et al. (2020)	USA	300	People with higher green self-identity perceived a higher value in PHA-based products.
Gill et al. (2020)	USA	218	Consumers moderately preferring eco-friendly disposable dinnerware were more likely to be influenced by the ‘compostability’ attribute.
Grebitus et al. (2020)	USA	346	When consumers were nudged with pro-environmental information, they opted for recycled and plant-based bottles and were willing to pay higher, focusing more on the raw material origin than end-of-life.
Marchi et al. (2020)	Italy	212	Most respondents preferred PLA bottles over PET and bio-PET over recycled PET.
Taufik et al. (2020)	Germany	281	Respondents attributed lower benefits to recyclable bioplastics and lowest to fossil-based plastics than compostable plastics.
Friedrich (2022)	Germany	356	Respondents were willing to pay more for bioplastic-based apparel products than their packaging.
Moshood et al. (2022c)	Malaysia	386	Environmental incentives are one of the reasons for respondents switching to biodegradable plastics.

LCA: life cycle assessments; PET: polyethylene terephthalate; PLA: polylactic acid; USA: United States.

**Figure 3.**
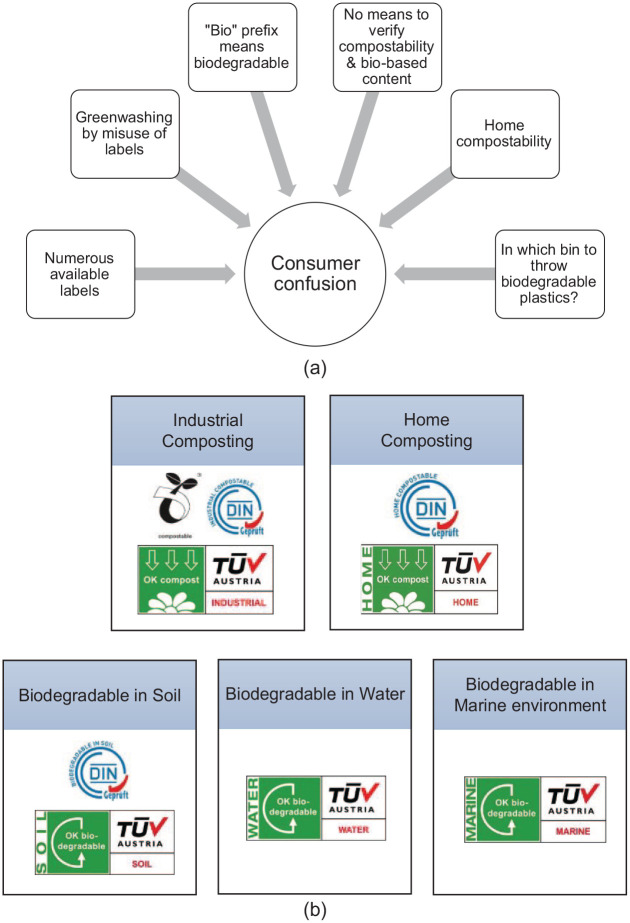
(a) Factors contributing to consumer confusion about biodegradable plastics. (b) Available labels in Europe for home and industrial compostability plus the labels for biodegradability in soil, water and marine environment. (Din Certco – TÜV Rheinland, 2022; Ruggero et al., 2019; Shah et al., 2008; TÜV AUSTRIA Belgium, 2022a, 2022b).

From these studies, it was observed that consumers are confused about biodegradable plastics. Figure 3(a) shows eight common confusing aspects of biodegradable plastics expressed by consumers in the studied scientific literature.

Consumers can identify biodegradable plastics with the help of labels. Bioplastics fall under resin identification code number ‘7’ in the ‘Other’ category (ASTM, 2021; Niaounakis, 2019). There are numerous labelling methods available, issued by different international organizations, such as DIN CERTCO, TÜV AUSTRIA Belgium, US Biodegradable Products Institute and Japan Bioplastics Association (Niaounakis, 2019). Figure 3(b) illustrates common labels used in Europe. Ideally, proper labelling and information about biodegradable plastics should ease confusion surrounding their proper disposal (Dilkes-Hoffman et al., 2019; Kakadellis et al., 2021; Sandhu et al., 2021; Taufik et al., 2020); however, multiple label types increase this confusion instead of reducing it (Gisi et al., 2022; Kakadellis et al., 2021; Lynch et al., 2017).

In some cases, these labels were misused for communicating misleading information (i.e. greenwashing) to consumers regarding environmental performance, such as biodegradability, without any substantial proof (Freitas Netto et al., 2020; Tateishi, 2018). Consumers were found to be willing to pay higher prices and shift to properly labelled biodegradable plastics (Confente et al., 2020; Wensing et al., 2020). However, this high willingness to pay along with their misunderstanding that all bioplastics are compostable, often made consumers susceptible to greenwashing (Arikan and Ozsoy, 2015; Atiwesh et al., 2021; Nazareth et al., 2019; Orset et al., 2017; Philp et al., 2013; Viera et al., 2020). For instance, there were concerns about companies misusing the ‘bio’ label, which caused problems for plastic pollution-related policies (Lynch et al., 2017). Thus, the aforementioned confusion and prevalent greenwashing often lead to incorrect disposal (Nazareth et al., 2019).

Sometimes, the incorrect conceptual understanding of the consumers contributed to this confusion. For instance, some literature found that there was a common false opinion that most bioplastics are biodegradable (Dilkes-Hoffman et al., 2019; Blesin et al., 2017; Niaounakis, 2019). Even the available labels failed to simplify the differentiation between the biodegradability and bio-based content of biodegradable plastics (Charlebois et al., 2022; Rujnić-Sokele and Pilipović, 2017; Walker and Fequet, 2023). In other cases, consumers often perceived the ‘bio’ prefix as made from bio-based raw materials (Blesin et al., 2017) and were also confused about home-compostability (Briassoulis et al., 2021). Consumers were also found to be sceptical about the compatibility of biodegradable plastics with existing infrastructure, as well as the definition and degree of biodegradability, and sometimes even viewing biodegradable plastics as contaminants (Kakadellis et al., 2021; Lynch et al., 2017). Lastly, the most common one is the ‘which-bin-to-select?’ dilemma, where the consumers are unsure about the best source separation option for biodegradable plastics (Dilkes-Hoffman et al., 2019; Lynch et al., 2017; Marchi et al., 2020; Patrício Silva, 2021).

### Waste processing and material utilization

Due to the dilemma surrounding source separation of biodegradable plastics, they could land in either packaging waste, biowaste or residual waste streams (Mhaddolkar et al., 2024). Currently, biodegradable plastics are mostly incinerated owing to their lower volume and carbon neutrality (Lorber et al., 2015; Niaounakis, 2019; van Roijen and Miller, 2022), which could either be the case when they are collected with residual waste or when they are in reject fraction from the material recovery facility when collected with packaging waste.

#### Packaging waste stream

Although collection with packaging waste is a pre-requisite to be recycled (except organic recycling), the presence of biodegradable plastics could contribute to the contamination of conventional plastic recycling streams. Their presence alters the mechanical properties of and causes premature failure in conventional plastics (Moshood et al., 2022a; Nagy et al., 2018; Rujnić-Sokele and Pilipović, 2017; Shamsuddin et al., 2017). For example, PLA is a major contaminant in recycled PET; it degrades at the processing temperatures of PET and causes cloudy PET bottles (Cornell, 2007; La Mantia et al., 2002; McLauchlin et al., 2014). In addition, adding plasticized starch and PLA in polyethylene (PE) and polypropylene (PP) strongly reduced elongation at the break while keeping modulus and tensile strength largely unaffected, whereas PET considerably lost its impact strength (Åkesson et al., 2021). Alternatively, PHA and PE furanoate presented no identified risk (Alaerts et al., 2018); however, their higher quantities in the future could be problematic for conventional plastics recycling (Kuciel et al., 2018). The main reason for this contamination could be attributed to ill-equipped sorting technology (Hasso von Pogrell, 2017). Thus, new types of bioplastics should be introduced in the market only after studying available recycling infrastructure and contamination possibilities of existing recyclates (Alaerts et al., 2018).

##### Sorting biodegradable plastics

Waste sorting is an essential stage for material recycling, which ensures the recovery of the recyclables from mixed waste (Åkesson et al., 2021; Gundupalli et al., 2017; Waggoner and Tudryn, 2020). Although different sorting methodologies, namely NIR and air separation can effectively sort biodegradable plastics (Niaounakis, 2019; van Roijen and Miller, 2022; Zhu and Wang, 2020), numerous sorting cycles will be needed to capture all market-available biodegradable plastics (Gisi et al., 2022; Moshood et al., 2022a). Additionally, increased volumes are essential to make a profitable economic investment in the dedicated sorting infrastructure (Shamsuddin et al., 2017).

Existing literature extensively studied the sortability of PLA from conventional plastics using NIR sorting technology. PLA was distinguished and sorted from PP, PET, high-density PE, low-density PE, linear low-density PE, thermoplastic polyurethane, and polyvinyl chloride (Bert Handschick et al., 2012; Chen et al., 2021; Mhaddolkar et al., 2022; Ulrici et al., 2013). In some cases, inline NIR-spectroscopy and chemometric methods were used to identify PLA concentrations in PET polymer melt during its processing, when 100% PLA removal was unsuccessful (McLauchlin et al., 2014). On the other hand, polyhydroxy butyrate (PHB) was successfully sorted out from mixed conventional plastics and PLA using NIR sorting (Mhaddolkar and Vollprecht, 2022). Thus, more research is needed to test the sortability of biodegradable plastics other than PLA. However, the use of NIR sorting for sorting other types of biodegradable plastics was quite sparse.

Furthermore, considerable literature was available on the use of NIR technologies for monitoring the biodegradable plastic properties and structural changes during their production (Dai et al., 2015; Li et al., 2020) and recycling (Beltrán et al., 2016, 2019). In other cases, NIR spectroscopy was employed to observe changes in PLA spectra with varied talc content (Amirabadi et al., 2018) and study the effect of photolysis on PLA crystallinity and PLA/PHB blend (Ishikawa et al., 2015).

##### Recycling

Extensive studies are available about the mechanical recycling of biodegradable plastics. For instance, PLA was observed to have higher mechanical-recycling potential (Soroudi and Jakubowicz, 2013), and up to 10 extrusion cycles, PLA waste could be used as an additive in a neat polymer (Żenkiewicz et al., 2009). However, industrial-level mechanical recycling of PLA is unavailable because of its lower volume and market availability of recyclates (Maga et al., 2019). Nevertheless, it was proposed that once the usage of PLA is increased, recycling will be preferred over composting (Niaounakis, 2019; Sherwood et al., 2016). Moreover, although mechanically recycled PLA was mostly downcycled (Payne et al., 2019), its recycling is still more cost-effective than other recovery methods (Badia and Ribes-Greus, 2016). It was found that blending PLA with Poly(3-hydroxybutyrate-co-3-hydroxyvalerate) (PHBV) prevented degradation and improved recyclability (Zembouai et al., 2014). Similarly, PLA/PHB blends retained properties after more extrusion cycles (11 cycles), as compared to their individual performance (PHB: 3 cycles, PLA: 10 cycles) (Payne et al., 2019). On the contrary, the mechanical properties of the PHB and polycaprolactone blend deteriorated after five extrusion cycles (La Mantia et al., 2002). PHA is unstable at very high temperatures, which makes their recycling difficult (Nandakumar et al., 2021; Rudnik, 2019).

On the other hand, chemical recycling is gaining momentum for biodegradable plastics (Feghali et al., 2020; van Roijen and Miller, 2022). Alcoholysis is considered the most promising method of chemical recycling of PLA depolymerization (Lamberti et al., 2020, 2021). Although catalytic recycling can be applied to both PLA and PHB, comparatively more research is conducted on the depolymerization of PLA (Feghali et al., 2020; Lee et al., 2022). Furthermore, Carné Sánchez and Collinson (2011) demonstrated that PLA could be selectively depolymerized from PLA–PET mixed waste using zinc acetate. Their research allowed selective depolymerization of target plastic from mixed plastic waste using solvolysis and a suitable catalyst while filtering the non-target unchanged plastic. However, like mechanical recycling, these studies were also conducted only on a lab scale. Recently, Lamberti et al. (2021) opined that the synergistic effect of conducting alcoholysis of PLA using two catalysts (zinc acetate dihydrate and 4-(dimethylamino)pyridine) could be explored at an industrial level because of the commercial availability and cost-effectiveness of the catalysts. However, for this to be viable at the industrial level, the total operating cost should be less than the acquired profit (Lamberti et al., 2021), which is a common challenge in the case of chemical recycling (Garcia and Robertson, 2017). Finally, the pyrolysis of PLA yielded a high gas yield, of 80%–90% (Skvorčinskienė et al., 2023).

#### Biowaste stream

Biodegradable plastics are often proposed as biowaste collection aids and are hailed as beneficial for efficiently collecting food waste (Bátori et al., 2018; Cazaudehore et al., 2022; Kakadellis and Harris, 2020; Kakadellis et al., 2021). Scholars have argued that throwing biodegradable plastics with food waste will simplify their waste management and reduce the confusion of consumers (Kakadellis et al., 2021; van Roijen and Miller, 2022). For instance, PHA could be collected with biowaste because of its better biodegradability (Nandakumar et al., 2021). Moreover, Edo et al. (2022) suggested that combining compostable plastics with door-to-door collection could reduce the influx of conventional plastic in biowaste. However, whether they undergo organic treatment or are ultimately sorted out and incinerated, is another issue.

##### Composting

Biodegradability is a significant attraction of biodegradable plastics (Babaremu et al., 2023; Calabrò and Grosso, 2018; Meeks et al., 2015). However, biodegradability is the property to biodegrade and compostability is the property to biodegrade in a given condition and time-period (Avella et al., 2001; European Environment Agency, 2020; Meeks et al., 2015; Paul-Pont et al., 2023). Biodegradable plastics need to comply with the existing standards and to be treated in existing composting infrastructure they need to degrade as fast as biowaste, which is seldom the case (Niaounakis, 2019).

The biodegradation time was found to be variable depending on different biodegradable plastics. For instance, PLA biodegradation time largely depends on product dimensions, where products with smaller dimensions have lower biodegradation time (Funabashi et al., 2009; Kawashima et al., 2021). In addition, fewer PLA degrading microorganisms are present in the environment compared to PHA and starch-based plastics, resulting in slower degradation time (García-Depraect et al., 2022; Lamberti et al., 2020; Mohee et al., 2008; Nandakumar et al., 2021). It was also found that PLA degrades faster in thermophilic temperature (>58°C, aerobically and anaerobically) than in ambient temperature (Adhikari et al., 2016; Haider et al., 2019); which indicated that PLA biodegradability is dependent on the suitable environment (Rujnić-Sokele and Pilipović, 2017; Yadav and Hakkarainen, 2021). On the other hand, numerous intra- and extra-cellular microorganisms are available to degrade PHB (Emadian et al., 2017; Roohi et al., 2018).

A contradiction was observed between the findings related to the impact of biodegradable plastics on the plant growth and soil quality. On one hand, the microbial community’s population and diversity increased due to PLA degradation (Karamanlioglu et al., 2017; Ong and Sudesh, 2016), potentially influencing plant growth, which remained stable (Kawashima et al., 2021; Liwarska-Bizukojc, 2022). Moreover, PHB was found to be harmless as well as able to be completely degraded and absorbed into the soil (Roohi et al., 2018). On the other hand, biodegrading PBAT mulch film resulted in delayed germination and stress effect on seedling growth (Gao et al., 2022; Liwarska-Bizukojc, 2021), with the presence of micro-bioplastic affecting soil and its biota (Chah et al., 2022; Muroi et al., 2016). In addition, Accinelli et al. (2022) demonstrated that even after deteriorating for 12 months at agricultural locations, the ultra-thin Mater-Bi (starch-blend) compostable plastic films persisted in soil (>2 mm) and damaged the crops by producing fungi. Moreover, additives were also found to affect biodegradability (Lambert and Wagner, 2017), and some of them were toxic and affected the crop germination index (Lu et al., 2023). However, more research is needed to study the effects of biodegradable plastics on soil (Mo et al., 2023; Zhou et al., 2023).

Furthermore, the existing literature also presents a conflicting view on the value derived from composting these plastics. Firstly, a composted material will only add value if it is humified (form humus-like material on biodegradation) and not when it is mineralized (completely breakdown into CO_2_ and water) (ÖWAV, 2021). ÖWAV (2021) claimed that biodegradable plastics almost degrade entirely into CO_2_ and water; thus, adding no value to compost. For instance, PLA mineralizes when industrially composted (Lamberti et al., 2020). Secondly, the EN 13432 standard states that >90% of biodegradable plastic should be converted into CO_2_ in 6 months (Hann et al., 2020; Soroudi and Jakubowicz, 2013), raising questions about whether humus is produced from these plastics in the end. Adding to it, it was found that the biodegradable plastics did not contribute sufficient nutrients to the compost (Ahsan et al., 2023). Thus, instead of acting as a replacement for mineral fertilizer, biodegradable plastics could function as soil structure building material (Detzel et al., 2013; García-Depraect et al., 2021). On the contrary, some literature stated that biodegradable plastics indeed result in compost/fertilizer (Folino et al., 2020; Kawashima et al., 2021; Khosravi-Darania and Buccib, 2015). Although the experiments conducted by Hermann et al. (2011a) demonstrated a humus formation, the other articles failed to do the same. In conclusion, from the existing literature, it is unclear whether humus is produced from composting.

On the other hand, there are specific concerns among compost facilities about biodegradable plastics. Firstly, they are hesitant about the biodegradable plastics influx (Calabrò and Grosso, 2018), as most biodegrade only in specific environments (Rujnić-Sokele and Pilipović, 2017). Compost facilities are also worried about the compost quality and biodegradation time (Meeks et al., 2015), and a possible increase in non-biodegradable plastics in biowaste due to consumer negligence and confusion (Umweltbundesamt, 2020). Thus, even if biodegradable plastics can be treated in compost facilities, they should first be accepted for treatment by these facilities.

##### Anaerobic digestion

The production of digestate and biogas in an anaerobic digestion process provides an added advantage over composting (Bátori et al., 2018; Hermann et al., 2011b; Siracusa et al., 2008); however, its performance is affected by the type of biodegradable plastics treated (Kale et al., 2007). Most biodegradable plastics degraded better in anaerobic than aerobic environments, where they were co-digested with other low-carbon-sourced biowaste (Abraham et al., 2021). For example, PHB degraded much better in an anaerobic environment than in an aerobic, but alkaline pre-treatment was required to improve PLA degradation (Bátori et al., 2018; Kriswantoro et al., 2023). However, Zaborowska et al. (2023) reported that pre-treatment of PLA did not affect its biodegradability, but rather reduced the degradation time. Starch-based plastics were degraded by 50% and PLA by 70% in soil with anaerobic digestion thermophilic treatment (Papa et al., 2023). Cellulose-acetate bioplastic degraded better in anaerobic digestion (50%–36%) than in composting (≃18%) (Gadaleta et al., 2023).

The presence of biodegradable plastics affected the methane yield and the digestate quality. For instance, compostable plastic bags reduced methane yield by 29.5% (than the control batch) in high-solids anaerobic digestion (Niknejad et al., 2023). On the other hand, Dolci et al. (2022) reported that anaerobic digestion of Mater-Bi samples with food waste increased methane production to 2%/mass unit of food waste, for the digestion of food waste alone. It was found that the 2 months of biodegradation time stipulated by anaerobic digestion standards is not enough for certain biodegradable plastics (Cazaudehore et al., 2022; Walker and Rothman, 2020), which necessitates updating these standards to include such plastics (Bátori et al., 2018; van Roijen and Miller, 2022). Certain inorganic additives affected the biodegradation time and were present in the digestate (Bracciale et al., 2023); thus, the quality of the digestate needs to be assessed. Additionally, the undigested plastic bags could be converted into soil-friendly biocrude via hydrothermal liquification (Niknejad et al., 2023). As a contradiction, Briassoulis et al. (2021) argued that the anaerobic digestion of biodegradable plastics produced an insignificant amount of biomass and methane.

## Part (ii): Existing legislation

### EU legislation

The 13 legislations were categorized into ‘Waste related’, ‘Consumer related’ and ‘Others’, and the findings are summarized in Table 3a. In the ‘Waste related’ category, eight legislations were reviewed, out of which four mentioned the disposal of biodegradable plastics. The EU Waste Framework Directive (2018) and the guidance for separate municipal waste collection (Dubois et al., 2022) mentioned that biodegradable packaging should be collected with biowaste. However, the new Proposal for a Regulation on PPW (Packaging and Packaging Waste) Directive (2022) and EU policy framework on bio-based, biodegradable and compostable plastics (2022) explicitly instructed that tea bags, stickers for vegetables and fruits, coffee pods and very light plastic carrier bags are allowed to be industrially composted without material recycling, thus implied to be collected with biowaste. On the other hand, other biodegradable packaging were instructed to undergo material recycling without affecting the recyclability of conventional plastics; thus, to be collected with plastic waste. The remaining four legislations in this category provided general information about biodegradable plastics, which is summarized in Table 3a.

**Table 3a.** Overview of the 13 EU legislations.

Category	EU legislation	Are biodegradable plastics mentioned?		Is there information about the disposal of biodegradable plastic waste?	
		Yes/No	If yes, what information is provided? If no, is there any other relevant information provided?	Yes/No	If yes, what information is provided?
Waste related (8 nos.)	EU Waste Framework Directive (2018)	Yes	– Aerobic/anaerobic digestion of the compostable packaging could be considered in the recycling rate when certain conditions are met.	Yes	**Biodegradable** and **compostable packaging**is to be collected with **biowaste**.
	EU Plastic’s Strategy (2018)	Yes	– Biodegradable plastics are proposed as a solution for marine pollution. – There are three requirements for biodegradable plastics: (1) uniform labelling, (2) LCA for identifying suitable applications; (3) prohibition of oxo-degradable plastics. – Biodegradable plastics could prove suitable for certain applications; however, proper labelling and waste collection plus treatment infrastructure is crucial. Biowaste collection bags are targeted applications. – For the consumer product applications to work, there must be an existing biowaste collection infrastructure.	No	–
	Guidance for separate municipal waste collection (Dubois et al., 2022)	Yes	– Compostable and biodegradable products are beginning to be used by companies as strategies for circular packaging.	Yes	**Biodegradable plastics** could be collected along with the **biowaste** provided they are certified compostable with proper labelling and do not deteriorate the quality as well as the biowaste value.
	Lightweight Plastic Carrier Bags Directive (2015)	Yes	– EU-wide uniform labels for biodegradable and compostable carrier bags are needed. – Consumers should be informed about the compostable properties of bags. – There should be a separate standard for home composting. – Oxo-degradable plastic bags should not be labelled as biodegradable.	No	–
	Single Use Plastic Directive (2019)	Yes	– A definition of biodegradable plastics is provided. Biodegradable plastics should be included in the adapted definition of plastics.	No	–
	Packaging and Packaging Waste Directive (2018)	Yes	– Different definitions of biodegradable and compostable plastics are provided. – Compostable biodegradable (and bio-based recyclable) packaging is promoted for renewable-sourced packaging use when its LCA is environmentally beneficial, resulting in reduced raw-material import. – Member states are required to set up proper collection infrastructure to capture packaging waste, which is unfulfilled for biodegradable plastics. – Aerobic/anaerobic digestion of the compostable packaging can be considered in the recycling rate when certain conditions are met.	No	–
	New Proposal for a Regulation on PPW Directive (2022)	Yes	– Annex-III enlisted conditions for compostable packaging: ○ its use should considerably increase biowaste collection and reduce compost contamination by non-compostable packaging (and not increase it) ○ it should be designed for treatment in a biowaste stream and be ‘biodegradable’ ○ it is chosen only after avoidance or use of reusable packaging is impossible	Yes	Instructed **four types of compostable plastic** items to be collected with **biowaste**. The **rest** should be sent for material recovery, thus to be collected with **plastic waste**.
	EU policy framework on bio-based, biodegradable and compostable plastics (2022)	Yes	– A comprehensive overview was provided of the advantages and challenges related to bio-based, biodegradable and compostable plastics, with a clear differentiation between compostability and biodegradability. – Biodegradable plastics could contaminate conventional-plastic recyclates, but proper labelling and consumer awareness through information campaigns (a bottom-up approach) could ensure proper disposal.	Yes	Instructed **four types of compostable plastic** items to be collected with **biowaste**. The **rest** should be sent for material recovery; thus, to be collected with **plastic waste**.
Consumer related (2 nos.)	New Consumer Agenda (2020)	No	– Better awareness should be created amongst the consumers with proper labelling and avoiding greenwashing.	No	–
	Protection against unfair practices and better information (2022)	No	– Consumers should be protected against greenwashing, early obsolescence practices and the use of non-transparent and misleading information tools and labels about sustainability.	No	–
Other (3 nos.)	Fertilising Products Regulations (2019)	No	– Biodegradable plastics used as packaging or for biowaste collection are not covered in the definition of ‘other allowed polymers’ in the EU fertilizing product, and hence should be included.	No	–
	Updated Bioeconomy Strategy (2018)	Yes	– Marine-biodegradable plastics are promoted as one of the replacement options (along with bio-based and recyclable plastics) for fossil-based non-biodegradable plastics and a measure to curb marine pollution. – These plastics have harmful effects on the environment and need to have a lifecycle view.	No	–
	Sustainable Carbon Cycles (2021)	No	– At least 20% of carbon used in plastic and chemical products should be sourced sustainably from non-fossil materials. – One of the three key actions is recycling carbon from waste streams to replace fossil carbon.	No	–

The two studied EU legislations in the ‘Consumer related’ category were mainly related to creating better awareness amongst consumers with proper labelling and avoiding greenwashing (New Consumer Agenda, 2020; Protection against unfair practices and better information, 2022). Lastly, in the ‘Others’ category, three EU legislations that could be related to biodegradable plastics were selected. It was found that the Fertilising Products Regulations (2019) did not include biodegradable plastics in the definition of ‘other allowed plastic’ in compost. Furthermore, the Updated Bioeconomy Strategy (2018) presented marine-biodegradable plastics as one of the replacement options for fossil-based non-biodegradable plastics in marine applications and as a measure to curb marine pollution. On the other hand, since after biodegradation >90% of carbon from biodegradable plastics is lost in the atmosphere as CO_2_ (Dimas, 2020; Soroudi and Jakubowicz, 2013), these plastics do not contribute to ‘recycling carbon from waste streams to replace fossil carbon’ key action mentioned in Sustainable Carbon Cycles (2021).

### National legislation and source separation guidelines

For the 13 countries, national legislation related to waste management and biowaste treatment along with source-separation guidelines for consumers provided by municipalities or public responsibility organizations were studied (Table 3b). Following is an overview of the studied literature.

**Table 3b.** National legislation and source separation guidelines for biodegradable plastics in 13 countries.

Countries	National waste legislations mention proper disposal		National legislation allowed them in biowaste treatment		Source separation guidelines
	Yes/No	If yes, where?	Yes/No	If yes, what?	
Austria	No	–	Yes	EN 13432 certified compostable plastics	**Plastic waste** or **residual waste**
Belgium	Yes (Partially)	Biodegradable bags – **Biowaste**	Yes	Biodegradable dustbin bags	**Residual waste**
Denmark	Yes	Biodegradable dustbin bags – **Biowaste** Other Biodegradable plastic packaging – **Plastic waste**	–	–	Biodegradable dustbin bags – **Biowaste** Other Biodegradable plastic packaging – **Plastic waste**
Finland	No	–	–	–	Biodegradable dustbin bags – **Biowaste** Other Biodegradable plastic packaging – **Plastic waste or mixed waste**
France	Yes (Partially)	Biodegradable bags – **Biowaste**	–	-	Biodegradable dustbin bags – **Biowaste** Other Biodegradable plastic packaging – **Plastic waste**
Germany	Yes	Certified biodegradable dustbin bags – **Biowaste** Other Biodegradable plastic packaging – **Plastic waste**	Yes	EN 13432 certified biodegradable bags qualify as biowaste	Plastic waste or residual waste
Ireland	No	–	–	–	**Plastic waste** Some parts allow in **Biowaste**
Italy	Yes	Biodegradable plastics – **Biowaste**	Yes	Biodegradable plastics	EN13432 certified biodegradable plastics: Food contaminated – **Biowaste** Uncontaminated – **Plastic waste**
Lithuania	No	–	–	–	Plastic waste
Luxembourg	No	–	–	–	Biodegradable dustbin bags – **Biowaste** Other Biodegradable plastic packaging – **Residual waste**
Netherlands	No	–	–	–	Biodegradable dustbin bags with Seedlings/OK Compostable – **Biowaste** Other Biodegradable plastic packaging – **Residual waste**
Slovenia	No	–	–	–	Biodegradable dustbin bags – **Biowaste** **No mention** of other Biodegradable plastic packaging
Sweden	No	–	–	–	Biodegradable dustbin bags – **Biowaste** **No mention** of other Biodegradable plastic packaging

References are given in the text below. The bold text signifies what information about the disposal pathways for biodegradable plastic is provided in the studied regulations.

LCA: life cycle assessments.

On studying the national legislations related to waste management of the 13 countries, it was found that only three (Denmark, Germany and Italy) countries provided information about the preferred disposal method of biodegradable plastics (DECRETO LEGISLATIVO 116/2020, 2020; Vejledning om sorteringskriterier for husholdningsaffald, 2022; Umweltbundesamt, 2020). In addition, two countries (Belgium and France) allowed biodegradable bags to be used for biowaste collection (Arrêté du 15 mars 2022, 2022; Code de l’environnement, 2022; Uitvoeringsplan huishoudelijk en gelijkaardig bedrijfsafval, 2020); however, there was no mention about other biodegradable plastic packaging. The national legislations of the Netherlands and Sweden asked for EN 13432 certified biodegradable plastics to be considered in extended producer responsibility regulation (Producentansvar för förpackningar, 2022; Verordening inzake producten voor eenmalig gebruik, 2022); however, their preferred disposal was not mentioned. Moreover, five countries (Austria, Finland, Lithuania, Luxembourg and Slovenia) only included definitions of biodegradable plastics, without mentioning their preferred disposal (Plastics Roadmap for Finland 2.0, 2022; PPW Law, 2022; Regulation on Packaging and Packaging Waste, 2022; Verpackungsverordnungs-Novelle 2021, 2021; Waste Management Law No. VIII-787, 2021). Lastly, they were not even mentioned in the Irish waste legislation (Single-use Plastics, 2021; Waste Action Plan for a Circular Economy, 2020).

Where available, the biowaste management-related national legislations were studied for the 13 countries. For four countries information was available about whether biodegradable plastics are allowed in compost facilities; Austria and Germany allowed only certified compostable dustbin bags (Federal Ministry for the Environment, Nature Conservation and Nuclear Safety (BMU), 2013; Kompostverordnung, 2001), Belgium allowed household biowaste collection bags, stickers for fruits and vegetables, and compostable cutlery at large events (Uitvoeringsplan huishoudelijk en gelijkaardig bedrijfsafval, 2020), whereas Italy allowed all kinds of biodegradable plastics (DECRETO LEGISLATIVO 116/2020, 2020). The other nine countries did not mention biodegradable plastics.

Studying the source separation guidelines of the 13 countries provided insight into where the consumers were instructed to throw biodegradable plastics and whether it coincided with the information provided in their national legislation. The Danish and Italian source separation guidelines matched their national legislations (amaROMA, 2021; CONAi, 2020; Vejledning om sorteringskriterier for husholdningsaffald, 2022). In the case of France, the guidelines provided information for the disposal of both biodegradable packaging and dustbin bags (Evolis 23, 2020; Municipalité de paris, 2019). Conversely, instructions for source separation guidelines in Austria, Germany and Belgium differed from their national legislations. Here, even when the national legislation permitted certified biodegradable dustbin bags in biowaste, the source separation guidelines instructed them to be disposed of in residual or plastic waste bins (Altstoff Recycling Austria AG, 2022; OVAM, 2022a, 2022b; Sametinger, 2017; viaco, 2021). Furthermore, for five countries (Finland, Lithuania, Luxembourg, Ireland and the Netherlands), these guidelines provided disposal information in absence of national legislation (Gamtos ateitis, 2020; Jäkälä, 2020; milieu centraal, 2022a, 2022b, 2022c; mywaste, 2021; Rinkiin, 2018; VANG Huishoudelijk afval, 2022; Ville de Luxembourg, 2022a, 2022b). While the Slovenian sorting guidelines mentioned only biodegradable dustbin bags and not packaging (Jeko, 2021; Saubermacher Komunala, 2022), Swedish sorting guidelines had no exclusive mention of biodegradable plastics (Du Är Hemma, 2022; Eskilstuna Energi and Miljö, 2018; Västblekinge Miljö AB, 2020).

## Challenges, opportunities and recommendations

### Identified challenges and opportunities

Throughout the review, multiple challenges in all the phases of biodegradable plastic waste management and related legislation were identified. And as ‘every challenge is an opportunity in disguise’, this provides a myriad of research opportunities that will aid in resolving the identified challenges.

The first identified challenge is the variety of biodegradable plastics available in the market. Biodegradable plastics represent about 52% of bioplastics on the market (European Bioplastics e.V., 2022). Alongside the increased production of certain biodegradable plastics, new kinds of these plastics are continuously developed. These different varieties pose a challenge in establishing an optimum waste management system to recover all types of bioplastics entering the market; similar to the challenge faced by the different varieties of flexible plastic packaging (Horodytska et al., 2018; Kurmayer, 2022). This, in other words, presents an opportunity to adhere to a few prominent types of biodegradable plastics in the market for suitable applications and set up a proper waste management infrastructure for them.

The waste collection phase also presented certain challenges. Although existing collection infrastructure can be used for collecting biodegradable plastic waste, where to collect it is not yet clear, which is also reflected on the consumer side. Although biodegradable plastics are promoted to improve food-waste collection (Kakadellis et al., 2021; van Roijen and Miller, 2022), this might have a rebound effect by increasing the conventional plastic influx in biowaste (Bátori et al., 2018; Mehta et al., 2021), because of improper disposal. Consumers often need clarification about appropriate disposal and are also prone to greenwashing from manufacturers. In some cases, products falsely display compostability labels. To combat this, the compostability certification companies published a ‘blacklist’ as a part of their quality policy, banning select labels due to misuse and greenwashing (TÜV AUSTRIA Belgium, 2022c). However, consumers need to be aware of this list to make informed choices. This necessitates better control over the sale of false-claim products in the market. In addition, companies should submit proof of their claims, termed ‘substantiating’ in the environmental consumer communication guide of European Bioplastics e.V. (2016).

Thus, communicating proper disposal methods is essential to accrue the environmental benefits of biodegradable plastics (Taufik et al., 2020), which could be achieved with clear labelling with location-based disposal information (Burrows et al., 2022; Wydra et al., 2017). In a nutshell, for an optimum waste collection of biodegradable plastics, establishing a suitable infrastructure, creating consumer awareness and preventing greenwashing is crucial. This provides a prospect to test novel ideas, like digital-product-passports (Regulation on Ecodesign for Sustainable Products, 2022) and digital watermarks (HolyGrail, 2023), and to see if they facilitate an informed disposal of biodegradable plastics. Moreover, the consumer confusion surrounding disposal was captured only with qualitative research, and not on their actual disposal behaviour. This area could also be explored as a future research possibility.

Compared to other biodegradable plastics, substantial literature was available on the NIR sorting of PLA (Bert Handschick et al., 2012; Chen et al., 2021; Mhaddolkar et al., 2022; Ulrici et al., 2013). However, more research is present on the use of NIR spectroscopy for observing the properties and structural changes of biodegradable plastic than their sorting. The NIR sorting machine producers affirmed that bio-based biodegradable plastics could be sorted; however, their low volume in waste makes it less economical (BMEL Research Alliance, 2017). Using only a few prominent biodegradable plastics for suitable applications will contribute to higher volumes and more cost-effective investment in waste management infrastructure.

Challenges were also identified in the waste processing and material utilization stage. There are considerable lab-based studies about the recycling of PLA, PHA/PHB and some starch blends; however, an industry-level study is yet to be undertaken. Industry-level feasibility studies, especially for mechanical and chemical recycling of biodegradable plastics (other than PLA) are lacking, which presents prospective research opportunities. However, this can be achieved only after sufficient market volume (and waste volume) of these plastics is available. Moreover, even though composting is the most proposed end-of-life method, there is a debate about the value it adds in comparison to incineration, and unclear and inconsistent information is available about compostability. Similarly, in the case of anaerobic digestion, there are concerns among the stakeholders about digestate quality and its subsequent effect on the soil (Kakadellis et al., 2021). In other words, though there is a technical recycling possibility (Pomberger, 2021), actual recycling is challenging in dynamic conditions (Folino et al., 2023) due to lack of suitable infrastructure (Wydra et al., 2021). Thus, the possibilities of material recovery and chemical fertilizer replacement still need to be fully exploited (Gisi et al., 2022; van Roijen and Miller, 2022). Lastly, a life cycle assessment of the most suitable treatment pathways could also shed light on the ‘where-to-throw’ biodegradable plastics dilemma.

While looking into the 13 EU legislation, it was observed that they primarily focused on the definitions of biodegradable plastics, their genuine environmental benefits, proper certifications with uniform labelling and suitable applications; in some cases, also hailed as a solution for unavoidable littering. Although some legislation hinted at possible collection with biowaste, it was not clearly defined. The latest PPW Directive proposal and EU policy framework on bio-based, biodegradable and compostable plastics have instructed four kinds of compostable plastic packaging to be organically recycled, whereas the other types of biodegradable packaging were directed to material recycling with the condition that they should not affect conventional plastic recycling. Although this is a considerable development in bioplastics-related legislation, diverting these biodegradable plastics into two different streams could cause more confusion than eliminating it. One solution would be to provide compostability labelling only for the packaging to be collected with biowaste, instead of all compostable packaging. And even if they are collected for material recycling, it is doubtful if they will be recycled or incinerated. In addition, these legislations excluded the discussion on the eco-friendliness of fossil-based compostable plastics. It will be effective if legislation pays more attention to better source-separation guidelines for biodegradable plastics, restricting the use of a few kinds of biodegradable plastic for certain applications, and environmental evaluation of fossil-based biodegradable plastics.

Lastly, on studying the 13 EU countries, it was observed that the disposal method proposed by source separation guidelines, which directly influences consumers, was different than the legislatively permitted disposal method in most countries. Moreover, some countries only defined biodegradable plastics. This definition failed to mention EN 13432 compostability certification and consider the presence of appropriate waste-collection infrastructure. Furthermore, most countries allowed biowaste collection with biodegradable bags, whereas biodegradable packaging was directed to plastic or residual waste. Confused consumers could be prone to improper sorting behaviour in cases with no mention of a preferred disposal method for biodegradable packaging. Informative and uniform labelling of plastic products is crucial to address consumer confusion, which should be supported by proper waste management infrastructure with adept national legislation and sorting guidelines.

### Recommendations

It is crucial to collect biodegradable plastics in relevant waste streams based on the most appropriate treatment method. For instance, unless biodegradable plastics are treated in organic recycling facilities (and not incinerated), they could be disposed of with plastic waste (instead of biowaste). As presently biodegradable plastics are mainly incinerated (Lorber et al., 2015; Niaounakis, 2019), their collection with plastic waste will ensure derivation of maximum energetic value (with comparatively lesser surface contamination than when sent for incineration after being collected in biowaste); and at the same time keep them available for material recovery when possible. In addition, collecting biodegradable plastics with plastic waste (and not residual waste) could decrease consumer confusion (about where-to-throw?) in the future, once the infrastructure for material recovery is in place. Additionally, the NIR sorting machines should be updated with the information about the market-available biodegradable plastics to reduce the contamination of conventional plastic recyclates. Nevertheless, this choice of collecting biodegradable plastics with plastic instead of residual waste needs to be evaluated using a life cycle assessment. On the other hand, if biodegradable plastics (only certified and proven compostable) are used to collect biowaste, they should be accepted and treated in the biowaste treatment facility.

Drawing from the above discussions, the following recommendations are provided for improved management of biodegradable plastic waste:

1. There should be a proper source separation guideline supported by national legislation for where to collect biodegradable plastics, which needs to be clearly communicated to consumers.
2. Consumers should be able to distinguish biodegradable plastics from non-biodegradable plastics.
3. If mechanical recycling is found to be a suitable waste treatment method, dedicated infrastructure should be available for the same.
4. In case the biodegradable plastics are to be treated in organic waste treatment facilities, the available infrastructure should accept these plastics.

## Conclusion

The present review provides a holistic overview of challenges and opportunities for biodegradable plastics waste management (mainly, biodegradable plastics as products, collecting biodegradable plastic waste, waste processing and material utilization) and a comprehensive summary of 13 EU laws and national legislations plus source separation guidelines of 13 countries. Although biodegradable plastics are perceived as an environmentally friendly alternative to conventional plastics, they have considerable challenges related to waste management. The following are the main findings:

- The low volumes and numerous varieties of biodegradable plastics make their waste management challenging.
- Biodegradable plastics are promoted as food-waste collection aids, but consumers are often confused about proper disposal and are prone to greenwashing from manufacturers. This indicates the need for suitable collection approaches, improving consumer awareness, and preventing greenwashing.
- Existing waste sorting infrastructure is applicable for biodegradable plastics; however, their low volume and different types make efficient recovery an expensive capital investment. In addition, near-infrared sorting of PLA is conducted extensively compared to other biodegradable plastics.
- Lab-scale studies of mechanical recycling of prominent biodegradable plastics have been conducted; however, industry-level studies demonstrating recycling on a full scale are unavailable. In addition, no clear and consistent information about compostability of biodegradable plastics exists.
- Although existing EU legislation briefly discusses biodegradable plastics, only the recent proposal on plastic packaging waste and the EU policy framework on bio-based, biodegradable and compostable plastics clearly address their disposal.
- Considerable confusion was observed at the legislative level in most studied countries, where there was no congruence between the available national legislation and the source separation guidelines.

Thus, optimally managing biodegradable plastic waste requires a systemic approach, where consumers, manufacturers, waste managers and legislators all play essential roles.

Lastly, certain gaps were identified in the existing literature, which provided a possibility for future research. Accordingly, three major ones are presented as follows:

- The sorting behaviour of consumers concerning biodegradable plastics needs to be studied, mainly, where they think they should throw biodegradable plastics and where are they throwing them.
- There are considerable ongoing greenwashing practices, for example, misuse of compostability labels on non-biodegradable plastic items (TÜV AUSTRIA Belgium, 2022c); thus, there is a need to study misleading products available in the market, and reasons for their circulation.
- With the wide variety of biodegradable plastics available in the market, there is a need to test the near-infrared sorting of biodegradable plastics other than PLA.
